# Specific Volumetric Weight-Driven Shift in Microbiota Compositions With Saccharifying Activity Change in Starter for Chinese Baijiu Fermentation

**DOI:** 10.3389/fmicb.2018.02349

**Published:** 2018-09-28

**Authors:** Bowen Wang, Qun Wu, Yan Xu, Baoguo Sun

**Affiliations:** ^1^Key Laboratory of Industrial Biotechnology of Ministry of Education, State Key Laboratory of Food Science and Technology, Synergetic Innovation Center of Food Safety and Nutrition, School of Biotechnology, Suqian Industrial Technology Research Institute of Jiangnan university, Jiangnan University, Wuxi, China; ^2^Beijing Advanced Innovation Center for Food Nutrition and Human Health, School of Food and Chemical Engineering, Beijing Technology and Business University, Beijing, China

**Keywords:** Chinese Baijiu, starter *Jiuqu*, functional microbes, volumetric weight, simultaneous saccharification and fermentation, high-throughput amplicons sequencing, metaproteomics

## Abstract

Chinese starter *Jiuqu*, traditionally produced by spontaneous fermentation and always squeezed into bricks, serves as a vital saccharifying agent for simultaneous saccharification and fermentation of Chinese Baijiu. It is important to reveal the key saccharifying microbiota and the driving force to improve the quality of *Jiuqu*. Here we studied the compositions of the microbiota by high-throughput amplicons sequencing analysis in *Jiuqu*, and revealed eight bacterial and seven fungal genera as the dominant community members. Among them, *Lactobacillus, Aspergillus, Pichia, Saccharomyces, Rhizopus* were the main contributors of proteins by metaproteomics analysis. Whereas, only *Lactobacillus, Pichia, Rhizopus* appeared as key actors for saccharification by secreting three glycosidases and two glycosyltransferases, and it indicated they were the key saccharifying microbiota in *Jiuqu*. Especially, *Rhizopus* secreted the most abundant glucoamylase. Interestingly, these three active genera significantly decreased and the key saccharifying enzymes were down-expressed, when *Jiuqu* was produced in diffused shape with a low volumetric weight. *Rhizopus microsporus*, the main producer of glucoamylase, was positively correlated with volumetric weight of *Jiuqu*. It indicated volumetric weight was the major driving force of the key saccharifying microbiota in *Jiuqu*. This work provides deep insights of key saccharifying microbiota, and indicates the main driving force for the key microbe. Furthermore, this finding can contribute to the improvement of saccharifying agent for food fermentation.

## Introduction

Simultaneous saccharification and fermentation widely exists in the production of foods and beverages (Li et al., 2013; Chen et al., 2014). This process contributes to metabolize macromolecules, generate various metabolites, and finally improve the quality and safety of foods and beverages via selecting available and efficient saccharifying agent (Singh and Soni, 2001; Soergel et al., 2012; Swain et al., 2013). Saccharifying agent is generally produced by spontaneous fermentation in the Orient (Zhu and Tramper, 2013). It’s important to understand the key microbiota and enzymes formation to control the quality of the saccharifying agent.

Chinese starter *Jiuqu* serves as the main saccharifying agent of the simultaneous saccharification and fermentation for Chinese Baijiu (Jin et al., 2017; Wang et al., 2017c; Liu and Sun, 2018). *Jiuqu* enriches various microorganisms including molds, yeasts, and bacteria from local environment (Jin et al., 2017). Several filamentous fungi, including *Aspergillus, Rhizopus, Paecilomyces*, could produce many saccharifying enzymes to degrade the starch material into fermentable sugar in the fermentation by the culture-dependent method (Chen et al., 2014). Whereas, the individual species maybe present the different metabolic characteristics in the complex communities, perhaps influenced by other species in a mixed-culture system (Kong et al., 2014). Subsequently, several metagenomic studies shed light on the structure *in situ* patterns of microbial communities in *Jiuqu*. These studies revealed the dominant bacterial and fungal strains in *Jiuqu*, including *Lactobacillus, Bacillus, Rhizopus, Aspergillus, Saccharomyces*, and some non-*Saccharomyces* genera (*Saccharomycopsis, Wickerhamomyces*, and *Pichia*) (Wang et al., 2008, 2011, 2017a,b; Gao et al., 2010; Xiu et al., 2012; Li H. et al., 2014; Gou et al., 2015; Su et al., 2015; Wang and Xu, 2015; Zheng X.W. et al., 2015). Totally, these studies provided an extended catalog of the catalytic potential microorganisms, however, these approach do not present direct information on the expressed genes and associated active metabolic pathways in *Jiuqu*. It’s a challenge to identify the key functional microbiota in a complex community only via the genomic information. Recently, metatranscriptomics and metaproteomics studies provided distinct insight into the active metabolic communities in the traditional fermented foods and beverages (Chen et al., 2017; Huang et al., 2017; Liu et al., 2017; Song et al., 2017). Fungi were the active community members and potential secreted key enzymes related to starch metabolism in the *Jiuqu* by metatranscriptomics approach (Huang et al., 2017). Moreover, several metaproteomics studies revealed fungi were the major host of microbial enzymes in wheat-*Qu* for Chinese rice wine (Zhang et al., 2012) and Pu-erh tea (Zheng et al., 2015). These studies proved that such meta-omics approaches were feasible to study the active community members and enzymatic characteristic of complex microbial communities.

In this study, we illustrated the microbial and enzymatic profiles of *Jiuqu* via combining high-throughput sequencing technologies (16S rRNA gene and internal transcribed space amplicon sequencing) and metaproteomics analysis. We revealed the key saccharifying microbiota *in situ* patterns of microbial communities and identified the driving force to influence the functional microbiota formation *in vitro* system. Furthermore, this work provides an available approach to identify the functional microbiota and establish the accurate association between active microorganism and key enzymes in foods and beverages fermentation.

## Materials and Methods

### Sample Collection

**FIGURE 1:**
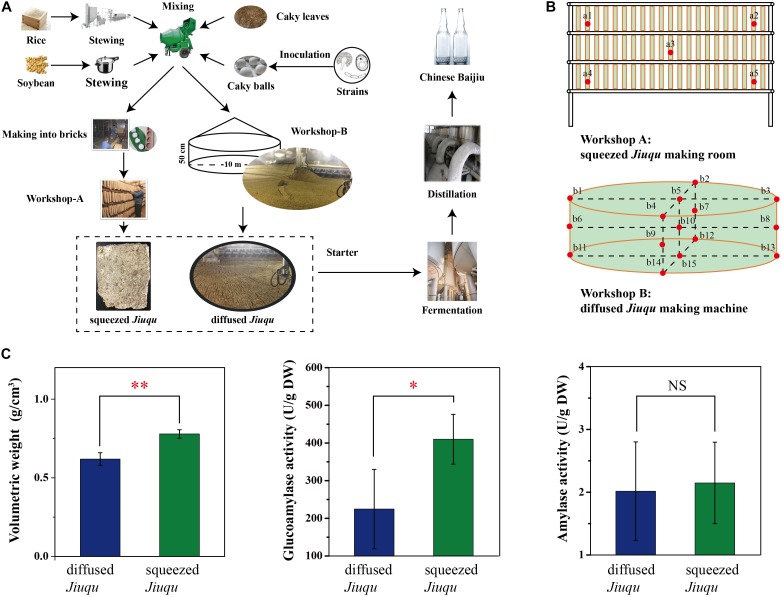
Chinese Chixiang Aroma-Type **(A)** starter *Jiuqu* and Baijiu production and **(B)** samples collecting from squeezed *Jiuqu* making room and diffused *Jiuqu* making machine, **(C)** physicochemical and enzymatic activity profiles of diffused and squeezed *Jiuqu* (paired-sample *t*-test: NS, no significant; ^∗^, *p* < 0.05; ^∗∗^, *p* < 0.01; ^∗∗∗^, *p* < 0.001).

*Jiuqu* samples were collected in September 2016 from a famous distillery in Guangzhou province, China (29.43 N, 115.59 E) (**Figure 1A**). Traditional *bingqu*, named squeezed *Jiuqu*, is developed in a *Qu*-making room. Modern mechanical-making moldy bran (*Fuqu*), named diffused *Jiuqu*, is prepared in a *Qu*-making machine. Compared to diffused *Jiuqu*, squeezed *Jiuqu* is made into bricks (**Figure 1A**). The sampling sites were shown in **Figure 1**. At each room, five *Jiuqu* blocks from upper, middle, and lower locations were collected, grinded, and mixed as one squeezed *Jiuqu* sample for per room (**Figure 1B**). Diffused *Jiuqu* samples were selected from five different sites in upper, middle, and lower locations in *Qu*-making machine (**Figure 1B**). Each *Jiuqu* group selected three paralleled samples. A 500.00 g per *Jiuqu* sample were harvested from the workshop and immediately frozen in liquid nitrogen, then kept on dry ice and stored in a -80°C freezer until analyses. Another 500.00 g per sample were kept on ice after harvested and stored in a 4°C freezer. Sample 1, 2, 3 was grouped for diffused *Jiuqu* and Sample 4, 5, 6 was grouped for squeezed *Jiuqu*.

### Physicochemical Parameters and Enzyme Activity Analyses

Volumetric weight of *Jiuqu* was based on the ratio of its mass and volume, and volume of *Jiuqu* was measured by displacement of water. Moisture content of *Jiuqu* was measured by detecting its weight loss after drying 10.00 g of sample at 105°C for four hours (sufficient to ensure constant weight). Acidity of *Jiuqu* was analyzed by acid-base titration. The glucoamylase and amylase activity of *Jiuqu* was measured as previous reports (Li H. et al., 2014; Li Z. et al., 2014). The unit of glucoamylase activity was defined as the amount (μg) of glucose converted from starch by one g of sample per minute under the assay conditions. The unit of amylase activity was defined as the amount (g) of liquefying starch by one g of sample per minute under the assay conditions. All analyses of samples are calculated by dry weight (DW).

### Total DNA Extraction, Amplification, and Sequence Processing

Total DNA of samples was extracted by the E.Z.N.A. (easy nucleic acid isolation) soil DNA Kit (Omega Bio-Tek, Norcross, GA, United States) according to the manufacturer’s instructions. For bacteria, the V3–V4 hypervariable region of the 16S rRNA gene was amplified by the universal primers of the forward 338F and the reverse 806R (Soergel et al., 2012). For fungi, the ITS was amplified by the primers of ITS1F and ITS2 (Buee et al., 2009). These primers contained a set of barcode sequences unique to each sample. PCR products were purified by a PCR purification kita PCR purification kit, and its concentrations were measured by the Thermo Scientific NanoDrop 8000 UV-Vis Spectrophotometer (NanoDrop Technologies, Wilmington, DE, United States). The barcoded PCR products were sequenced by a MiSeq benchtop sequencer for 250-bp paired-end sequencing (2 bp × 250 bp; Illumina, San Diego, CA, United States) at Beijing Auwigene Tech, Ltd. (Beijing, China). All sequences generated data were submitted in the DDBJ database under accession numbers of DRA006799 and DRA006800.

The Miseq-generated raw sequence data were processed by QIIME pipeline (v 1.8.0) (Caporaso et al., 2010). Quality trimming was conducted by removing the sequences with quality scores <30. Further analysis was based on choosing only sequences over 200 bp. The sequences were removed that did not perfectly match the PCR primer, had non-assigned tags, or had an N base. Chimeras were removed by UCHIME software (Edgar et al., 2011). Then, the trimmed sequences were clustered into operational taxonomic units (OTUs) with a 97% identity threshold by Qiime’s uclust pipeline (Edgar, 2010). A single representative sequence from each OTU was aligned to the Greengenes database (v13.8) and the UNITE fungal ITS database (v6.0) (Koljalg et al., 2005; DeSantis et al., 2006). Singleton OTUs were removed before further analysis. Chao1 richness and Shannon diversity indexes were calculated by QIIME (v 1.8.0) (Tao et al., 2014).

### Real-Time Quantitative PCR (qPCR)

Populations of different microbes were determined by qPCR in *Jiuqu*. These assays were carried out by an Applied Biosystems StepOne real-time PCR platform (Applied Biosystems, Foster City, CA, United States) with a commercial kit (SYBR Premix ExTaqTM II, Takara, Dalian, China) according to the manufacturer’s instructions. The total genomic DNA was used as the template to amplify bacteria by primers 340F, 758R (Juck et al., 2000), molds by primers Fnpstr, Rnpstr (Rodriguez et al., 2012), yeasts by primers YEASTF, YEASTR (Bokulich et al., 2013). The calibration curves and amplification conditions were constructed as the previous study (Liu et al., 2017).

### Protein Preparation and Mass Spectrometry Analyses

To increase the reliability and achieve adequate predictive power for proteomic profiling of *Jiuqu*, three biological replicates per group were performed to global label-free quantitative proteomics analysis. The proteome sample preparation was detailed described as a previous study (Zhao et al., 2015) at Shanghai Majorbio Bio-pharm Technology Co., Ltd. (Shanghai, China). The samples were overnight digested by trypsin (1:100 w/w, trypsin to protein, Promega, Madison, WI, United States) at 37°C. Then the tryptic peptides were desalted by a Sep-Pak C18 cartridge (Waters, Milford, MA, United States) and dried in a vacuum concentrator. The peptides were quantitated by the Thermo Fisher Scientific Peptides Quantitation Kit (Thermo Fisher Scientific, Shanghai, China).

The peptides were analyzed by an Easy-nLC1200 coupled to a Q-Exactive mass spectrometer (Thermo Fisher Scientific, Bremen, Germany). Five microliters (approximately 7 μg total peptide) was loaded into a C18 analytical column (75 μm ID × 25 cm) (Thermo Fisher Scientific, Bremen, Germany). Mobile phase A (2% acetonitrile and 0.1% formic acid in HPLC water) and mobile phase B (80% acetonitrile and 0.1% formic acid in HPLC water) were established different gradients as follows: starting with 2 min of 6% B, 98 min of 23% B, 30 min of 29% B, 17 min of 38% B, 6 min of 100% B, followed by 20 min of 0% B. The flow rate was 300 nL/min. During the entire chromatographic process, the linear trap quadruple (LTQ) mass spectrometer was operated in a data-dependent MS/MS mode with the following parameters: DDA (MS m/z range: 350–1300), resolutions (70,000 at m/z 200). The 15 most intense precursors were selected for higher-energy collisional dissociation (HCD) fragmentation.

### Mass Spectrometric Data Analysis

Raw data were processed for database searching by Thermo Scientific^TM^ Proteome Discoverer^TM^ (PD) 1.4 software, connected to an in-house Mascot server (V 2.4.1, Matrix Science, Boston, MA, United States). Proteins were searched by the UniProt database^1^. The detailed procedure was described as the previous study (Cheow et al., 2016). The highest score for a given peptide mass (best match to that predicted in the database) was used to identify parent proteins. Gene Ontology (GO), Clusters of Orthologous Groups (COG), and Kyoto Encyclopedia of Genes and Genomes (KEGG) annotations for the identified proteins were assigned to the UniProt database. All protein group generated data were submitted in the iProX database^2^ under the project number of IPX0001228000.

### Data Analysis

Principal component analysis (PCA) of the community structure was analyzed by SPSS Statistics 19.0 (IBM^®^SPSS^®^ Statistics, NY, United States). Paired-Sample *t*-test was conducted to compare community structure, physicochemical parameters, enzyme activity between the two groups of samples. One-way analysis of variance (ANOVA) was conducted to compare contents, physicochemical parameters, enzyme activity among the single-strain cultivation. To analyze the relationships between microbial communities and exogenous factors, we calculated all possible Spearman’s rank correlations among the abundant genera (with average abundance of >1.00%). A network was created by Gephi (Web Atlas, Paris, France) to sort through and visualize the correlations.

### Fungal Strains Isolation and Cultivation

The fungal strains were isolated by Potato dextrose agar medium (PDA) (6.00 g/L potato extract, 20.00 g/L glucose, 0.20 g/L chloramphenicol, and 20.00 g/L agar) from *Jiuqu*, and filamentous fungi were enumerated by different colony morphologies (diameter, reverse side color, surface color, and spores) (Lv et al., 2012).

Genomic DNA of the single isolated strains was extracted by the instruction of the TIANamp DNA Kit (TIANGEN, Beijing, China). The internal transcribed spacer (ITS) region was amplified by primers ITS1 and ITS4 as the previous study described (White et al., 1990). DNA sequencing of the PCR products was conducted by Sangon Biotech (Shanghai) Co., Ltd. (Shanghai, China). The isolated strain was identified by blasting against the sequences^3^.

The identified filamentous fungus, *Rhizopus microsporus* JJ01, was isolated from *Jiuqu* samples. The genome sequence of JJ01 was deposited in GenBank under the project number of MH782032. Rice was steeped in water at 25°C for 10 h, then steamed at 121°C for 40 min, and inoculated with *R. microsporus* at 10^6^ spores/g level. Non-inoculated media were set as the control group. One hundred g per inoculated rice were transferred to 500 mL vessels and squeezed to different volumes, fermented at 30°C for 7 days. Samples were collected from each vessel at seventh day for physicochemical parameters, real-time quantitative PCR and enzyme activity analysis.

## Results

### Physicochemical and Enzymatic Activity of Diffused and Squeezed *Jiuqu*

Volumetric weight, glucoamylase, and amylase activity parameters were applied to describe the intrinsic and saccharifying characteristics of *Jiuqu* (**Figure 1**). As made into bricks, squeezed *Jiuqu* presented a higher volumetric weight (0.78 ± 0.03 g/cm^3^), and its glucoamylase and amylase activity reached 409.00 ± 66.03 and 2.15 ± 0.65 U/g DW (**Figure 1C**). When *Jiuqu* was produced in diffused shape, volumetric weight of diffused *Jiuqu* decreased to 0.62 ± 0.04 g/cm^3^ and glucoamylase and amylase activity decreased to 224.50 ± 105.20 and 2.02 ± 0.79 U/g DW (*p* < 0.05) (**Figure 1C**).

### Microbial Profiles of Diffused and Squeezed *Jiuqu*

**FIGURE 2:**
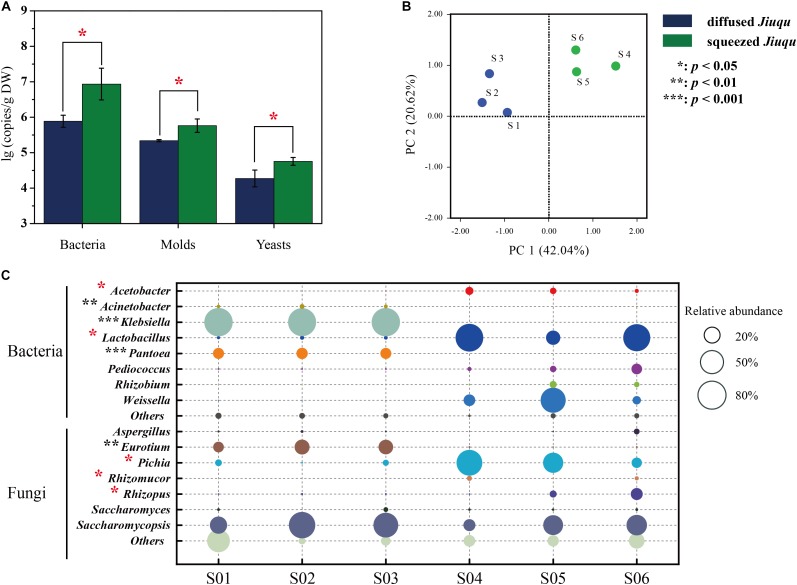
Microbiota components analysis across all the samples. **(A)** The contents of bacteria, molds and yeasts were analyzed by RT-qPCR. **(B)** Amplicons analysis represented the similarities of microbial compositions based on principal component analysis (PCA). **(C)** The different microbiota content between diffused and squeezed *Jiuqu* represented in bacterial and fungal distribution. Compared to diffused *Jiuqu*, red and black asterisks denote statistically significant increase and decrease in squeezed *Jiuqu* (paired-sample *t*-test: ^∗^, *p* < 0.05; ^∗∗^, *p* < 0.01; ^∗∗∗^, *p* < 0.001).

Microbial profiles of diffused and squeezed *Jiuqu* are shown in **Figure 2**. Bacteria, molds and yeasts reached 6.93 ± 0.45, 5.76 ± 0.19, 4.76 ± 0.11 lg (copies/g DW) in squeezed *Jiuqu*, whereas, those decreased to 5.89 ± 0.18, 5.34 ± 0.03, 4.23 ± 0.24 lg (copies/g DW) in diffused *Jiuqu* (*p* < 0.05) (**Figure 2A** and **Supplementary Table S1**).

Then, microbial community structure was studied via 16S rRNA and ITS sequence analysis (**Supplementary Figure S1** and **Table 1**). We obtained 56, 837 reads (18,946 ± 4,633 per sample) and 121, 338 reads (40, 446 ± 11, 580 per sample) in 16S rRNA and ITS sequences after quality control from squeezed *Jiuqu*. In addition, 16S rRNA and ITS gene amplicons sequences were clustered into 31 and 172 OTUs. The good coverage for 16S rRNA and ITS genes were above 98.0% (**Table 1**), and this indicates adequate sequencing depth of samples and members in microbial communities. Therefore, bacterial and fungal diversity analysis were conducted with the sequences normalized to 15, 730 and 23, 463 reads (**Supplementary Table S2**). Furthermore, high-throughput amplicons sequencing analysis revealed five bacterial and seven fungal genera were abundant in squeezed *Jiuqu* (relative abundance above 1.00%) (**Supplementary Figure S1A** and **Supplementary Table S3**). Especially, the bacterial genera *Lactobacillus, Weissella, Acetobacter, Pediococcus* and the fungal genera *Pichia, Saccharomycopsis, Rhizopus, Rhizomucor* were dominant in squeezed *Jiuqu* (**Supplementary Figures S1A,B** and **Supplementary Table S3**). Compared to squeezed *Jiuqu*, we obtained 65, 718 reads (21, 906 ± 2, 876 per sample) and 88, 551 reads (29, 517 ± 9, 767 per sample) in 16S rRNA and ITS sequences from diffused *Jiuqu*. In addition, 16S rRNA and ITS gene amplicons sequences were clustered into 29 and 159 OTUs. High-throughput amplicons sequencing analysis revealed four bacterial and five fungal genera were abundant in diffused *Jiuqu* (relative abundance above 1.00%) (**Supplementary Figure S1A** and **Supplementary Table S3**). Especially, the bacterial genera *Klebsiella, Pantoea, Acinetobacter* and the fungal genera *Saccharomycopsis, Eurotium, Cyberlindnera, Pichia* were dominant in diffused *Jiuqu* (**Supplementary Figures S1A,B** and **Supplementary Table S3**).

**Table 1:** Bacterial and fungal microbiota diversity index based on 16S rRNA and ITS amplicons sequencing across samples.

ID	Group	Clean reads		OTU number		Goods’ coverage	Chao1 richness		Shannon diversity index	
						
		Mean ± SD	*p*	Mean ± SD	*p*	Mean ± SD	Mean ± SD	*p*	Mean ± SD	*p*
Bacteria	Diffused *Jiuqu* (*n* = 3)	21,906 ± 2,876	0.40	24 ± 5	0.62	0.9990 ± 0.0004	77.9 ± 38.3	0.66	1.30 ± 0.09	0.09
	Squeezed *Jiuqu* (*n* = 3)	18,946 ± 4,618		22 ± 4		0.9990 ± 0.0001	66.8 ± 11.4		1.25 ± 0.32	
Fungi	Diffused *Jiuqu* (*n* = 3)	29,517 ± 9,767	0.28	76 ± 58	0.51	0.9943 ± 0.0036	392.8 ± 265.9	0.28	2.72 ± 1.65	0.75
	Squeezed *Jiuqu* (*n* = 3)	40,446 ± 11,580		106 ± 44		0.9990 ± 0.0001	392.8 ± 72.1		3.07 ± 0.74	


*The P-value was corrected with different group of samples by paired-sample t-test.*

Principal coordinates analysis was applied to evaluate similarities and differences in the prokaryotic and eukaryotic microbiota between diffused and squeezed *Jiuqu* (**Figure 2B**). Although the bacterial and fungal diversity showed no significant differences (*p* > 0.05) (**Table 1**), the compositions of microbial communities varied between two type *Jiuqu* (**Figure 2C** and **Supplementary Table S3**). *Acetobacter, Lactobacillus*, and *Weissella* reached 4.31 ± 2.45%, 56.49 ± 31.47%, 27.89 ± 30.48% in squeezed *Jiuqu*, whereas these genera decreased to 0.07 ± 0.03%, 1.43 ± 0.34%, 0.14 ± 0.02% in diffused *Jiuqu* (*p* < 0.05) (**Figure 2C** and **Supplementary Table S3**). Meanwhile, *Pichia, Rhizomucor*, and *Rhizopus* reached 39.79 ± 28.11%, 1.64 ± 1.27%, 6.72 ± 7.29% in squeezed *Jiuqu*, and these genera decreased to 2.98 ± 2.34%, 0.07 ± 0.01%, 0.38 ± 0.11% in diffused *Jiuqu* (*p* < 0.05) (**Figure 2C** and **Supplementary Table S3**).

### Enzymatic Profiles of *Jiuqu* by Metaproteomics Analysis

**FIGURE 3:**
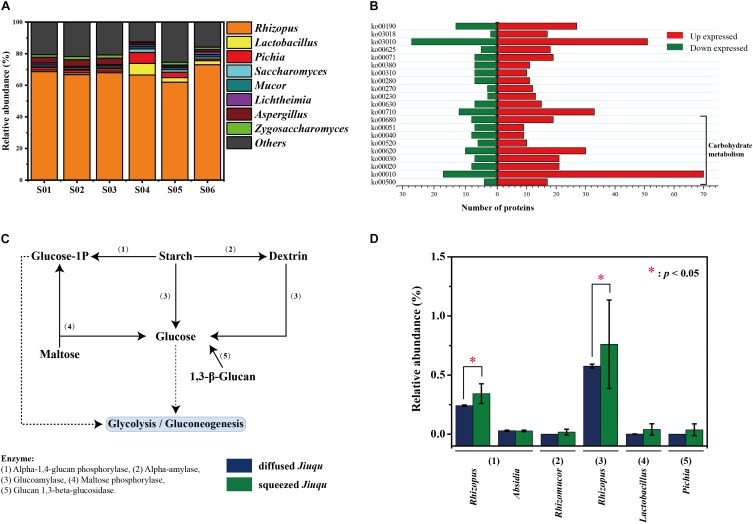
Enzymatic profiles of diffused and squeezed *Jiuqu*. **(A)** Taxonomy distribution of diffused and squeezed *Jiuqu* (*n* = 6, each bar *n* = 3). **(B)** The number of significantly different expressed proteins between diffused and squeezed *Jiuqu* by KEGG annotation. **(C)** The dominant saccharifying process in *Jiuqu* starter. **(D)** The core enzymes and microbiota related to the saccharifying process in *Jiuqu* starter. Compared to diffused *Jiuqu*, red asterisks denote statistically significant increase in squeezed *Jiuqu* (paired-sample *t*-test: ^∗^, *p* < 0.05; ^∗∗^, *p* < 0.01; ^∗∗∗^, *p* < 0.001).

To study enzymatic profiles of *Jiuqu*, we established a label-free quantitative proteomics approach on six samples, and all the data sets were combined and analyzed together (**Figure 3A**). Among 343, 096 spectra obtained, 49, 799 (14.51%) identified spectra were assigned to 10, 005 peptides and 3, 973 proteins. After filtering, we retained a total of 1, 733 potentially non-redundant protein groups (**Table 2** and **Supplementary Tables S4, S5**). A total of 1,421 identified proteins were associated with fungi spanning 8 phyla and 110 genera, and 183 identified proteins were associated with bacteria spanning 16 phyla and 58 genera (**Figure 3A** and **Supplementary Table S6**). Among them, five bacterial or fungal genera (the genus *Lactobacillus, Aspergillus, Pichia, Saccharomyces, Rhizopus*) were the main contributors of proteins, based on the total content of protein affiliated to the specific microbe (relative abundance above 1.00%). The genus *Rhizopus* (above 60.00%) were the most represent in all six samples. The genus *Lactobacillus* (4.27 ± 2.66%) was the second dominant microorganism in squeezed *Jiuqu*, whereas, this place was *Aspergillus* (3.95 ± 0.30%) in diffused *Jiuqu* (**Figure 3A** and **Supplementary Table S6**). According to Kyoto Encyclopedia of Genes and Genomes (KEGG) annotations, a number of 1,226 proteins were classified into 117 pathways (**Supplementary Table S7**).

**Table 2:** Overview of the metaproteomics results in Diffused and Squeezed *Jiuqu*.

Sample	Total	Different expressed proteins	Up expressed (squeezed/diffused *Jiuqu*)	Down expressed (squeezed/diffused *Jiuqu*)	Only in diffused *Jiuqu*	Only in squeezed *Jiuqu*
Diffused and squeezed *Jiuqu* (*n* = 6)	1,733	591	92	26	142	331


Among 1733 non-redundant protein groups, the expression of 591 proteins were significantly different between diffused and squeezed *Jiuqu* (*p* < 0.05 and fold change >1.20 or <0.87). The 331 protein groups were only identified and 92 non-redundant protein groups were up-expressed in squeezed *Jiuqu*, whereas, 42 protein groups were only identified and 26 non-redundant proteins groups were up-expressed in diffused *Jiuqu*. (**Table 2** and **Supplementary Table S5**). These 591 proteins were classified into 75 pathways by KEGG annotation, mainly related to carbohydrate metabolism, energy metabolism, translation, and amino acid metabolism (**Figure 3B**). Compared to diffused *Jiuqu*, the up-expressed proteins in squeezed *Jiuqu* were mainly related to carbohydrate metabolism, including starch and sucrose metabolism (ko00500), glycolysis/gluconeogenesis (ko00010), pentose phosphate pathway (ko00030), citrate cycle (ko00020), pyruvate metabolism (ko00620) and so on (**Figure 3B** and **Supplementary Table S7**).

Several saccharifying enzymes were identified in diffused and squeezed *Jiuqu* (**Figure 3C**). **Figure 3C** shows that alpha-1,4-glucan phosphorylase (EC 2.4.1.1), alpha-amylase (EC 3.2.1.1), glucoamylase (EC 3.2.1.3), maltose phosphorylase (EC 2.4.1.8), glucan 1,3-beta-glucosidase (EC 3.2.1.58) contributed to polysaccharides and oligosaccharides degradation. Glucoamylase and alpha-1,4-glucan phosphorylase were the top two abundant enzymes and attributed to the genus *Rhizopus* in *Jiuqu* (**Figure 3D**). The expression of glucoamylase and alpha-1,4-glucan phosphorylase reached 0.76 ± 0.46% and 0.34 ± 0.08% in squeezed *Jiuqu*, whereas, they decreased to 0.58 ± 0.02% and 0.24 ± 0.01% in diffused *Jiuqu* (*p* < 0.05) (**Figure 3D**).

### Key Functional Microbiota Showed a Significant Correlation With Volumetric Weight

**FIGURE 4:**
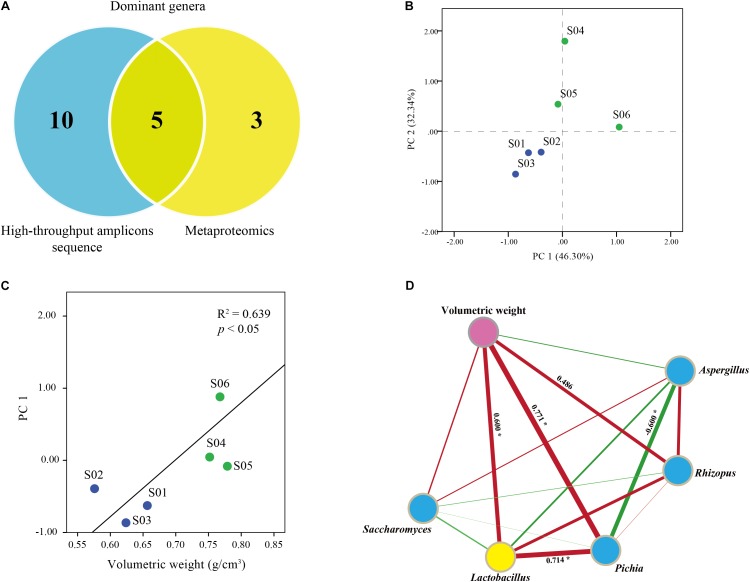
**(A)** Core functional microbes identified in diffused and squeezed *Jiuqu*. **(B)** Amplicons analysis represented the similarities of core functional microbes based on principal component analysis (PCA). **(C,D)** Correlation network between microbiota and volumetric weight factor.

We combined high-throughput amplicons and metaproteomics analysis to identify the key functional microbiota in *Jiuqu* (**Figure 4**). Hereon, high-throughput amplicons analysis revealed eight bacterial and seven fungal genera were abundant in *Jiuqu*. Among them, five bacterial or fungal genera (*Lactobacillus, Aspergillus, Pichia, Saccharomyces, Rhizopus*) was the main contributors of proteins by metaproteomics analysis (**Figure 4A**). Diffused and squeezed *Jiuqu* was classified into two clusters based on the amounts of the key functional microbiota (**Figure 4B**). Volumetric weight, the major exogenous factor, showed a significant correlation with the microbial factors (*R*^2^ = 0.64, *p* < 0.05) (**Figure 4C**). Then, we explored the correlations of microbes and volumetric weight based on Spearman’s rank correlations in *Jiuqu* (**Figure 4D**). *Lactobacillus, Pichia, Rhizopus*, the main saccharifying microbiota, were positively correlated with volumetric weight factor (Spearman’s | ρ| > 0.4, *p* < 0.05).

### Volumetric Weight Drove the Key Functional Microbiota

**FIGURE 5:**
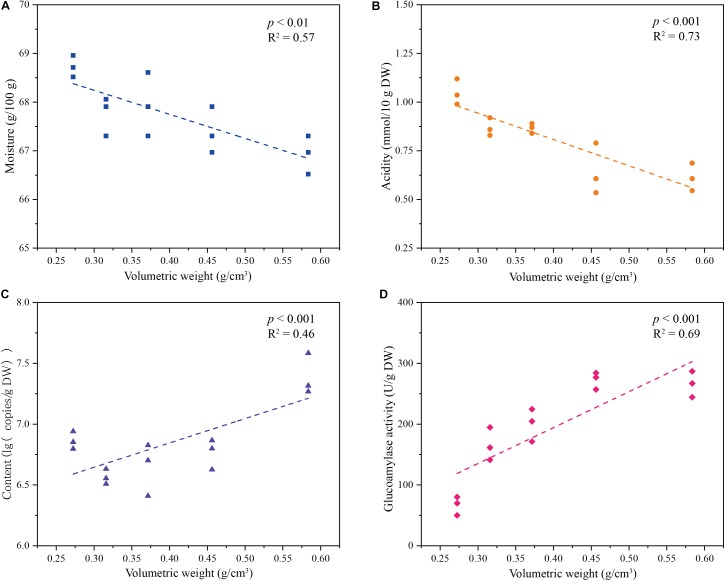
Dynamics of **(A)** moisture, **(B)** acidity, **(C)** microbial population, and **(D)** glucoamylase activity in Single-strain *Jiuqu* under different volumetric weight gradients.

We determined the impact of volumetric weight on the metabolism of *Rhizopus*, the main producer for key saccharifying enzymes (**Figure 5**). We set a series of volumetric weight gradient (from 0.27 ± 0.02 to 0.58 ± 0.01 g/cm^3^) to cultivate a single-strain *R. microsporus*, and then explored the dynamics of physicochemical index, microbial population, enzyme activity (**Figure 5** and **Supplementary Table S8**). As volumetric weight increased, moisture of the single-strain starter decreased from 68.73 ± 0.22 to 66.93 ± 0.39 g/100 g, and acidity decreased from 1.05 ± 0.07 to 0.61 ± 0.07 mmol/10 g DW (*p* < 0.05) (**Figures 5A,B**), whereas, the content of *R. microsporus* increased from 6.86 ± 0.07 to 7.39 ± 0.17 lg (copies/g DW), and glucoamylase activity increased from 66.69 ± 15.28 to 266.00 ± 21.30 U/g DW (*p* < 0.05) (**Figures 5C,D**). In addition, moisture and acidity indexes were negatively correlated with the volumetric weight of samples (*R*^2^ = 0.57, *p* < 0.01 and *R*^2^ = 0.73, *p* < 0.001), however, the content of the key saccharifying microbe and glucoamylase activity were positively correlated with the volumetric weight of samples (*R*^2^ = 0.46, *p* < 0.001 and *R*^2^ = 0.69, *p* < 0.001) (**Figure 5**).

## Discussion

In Chinese Baijiu production, starter *Jiuqu* provides the key saccharifying microbiota and enzymes to the fermentation process, contributing to polysaccharides degradation and flavor formation (Wu et al., 2009; Zheng et al., 2011). The compositions and amounts of microbial members determine the stability of productivity in *Jiuqu*. In addition, local exogenous and endogenous factors might strongly influence microbial community formation and metabolism in *Jiuqu*, including manufacture change, oxygen concentration, temperature change, heat transfer, moisture, or acidic stress (Li et al., 2016; Song et al., 2017; Xiao et al., 2017). It’s important to identify the key functional microbiota and the main driving force to regulate microbial formation in the saccharifying agent.

In this study, we determined the key saccharifying microbiota in *Jiuqu* by combining high-throughput amplicons sequencing and metaproteomics approaches. Interestingly, compared to squeezed *Jiuqu*, glucoamylase activity significantly decreased when *Jiuqu* was produced in diffused shape (**Figure 1C**). For microbial profiles of *Jiuqu*, bacteria, molds, yeasts significantly decreased in diffused *Jiuqu* (*p* < 0.05) (**Figure 2A** and **Supplementary Table S1**). Especially, the amount of the bacterial genera *Acetobacter, Lactobacillus* and the fungal genera *Pichia, Rhizomucor, Rhizopus* decreased significantly, although microbial diversity showed no significantly change in diffused *Jiuqu* (**Table 1**, **Figure 2C**). Furthermore, five saccharifying enzymes from *Lactobacillus, Pichia, Rhizopus* were down-expressed in diffused *Jiuqu* (**Figure 3**), consistent with a lower glucoamylase activity of diffused *Jiuqu*. We deduced that *Lactobacillus, Pichia, Rhizopus* mainly drove the saccharifying activity of *Jiuqu*.

The taxonomic distribution of all protein groups showed the presence of a few dominant microbial groups, including *Rhizopus, Lactobacillus, Pichia, Aspergillus*, and *Saccharomyces* (**Figure 3** and **Supplementary Table S6**). Fungi were the most abundant and active members, consistent with the functional microbiota in Chinese Nong-Flavor liquor starter (Huang et al., 2017). Especially, *Rhizopus*, the most dominant fungus, secreted glucoamylase playing a vital role in saccharifying agent (**Figure 3D**). This mesophilic genus is known for a high activity of glucoamylase and alpha-amylase (Almeida et al., 2017). Whereas, *Rhizopus* only had a high activity of glucoamylase in this study, distinguished with other agro-industrial wastes biotransformation process (Fernandez Nunez et al., 2017). In addition, other key saccharifying enzymes were affiliated to *Lactobacillus, Pichia, Rhizomucor*. This synergistic effect in complex microbial communities may be more beneficial to present a stable saccharifying efficiency. Further studies should develop synergistic effect on the function formation of microbial communities. Meanwhile, bacteria and yeasts could secrete various glycosidases to improve the aroma characteristics of the fermented foods and beverages (Zhao et al., 2015; Di Cagno et al., 2016; Padilla et al., 2016; Cappello et al., 2017). We found that specific bacteria and yeasts members took part in saccharifying activity in *Jiuqu*. Further, the role of glycosidases from bacteria and yeasts remains explored in Chinese Baijiu fermentation.

We also found the dominant microorganisms were different between high-throughput amplicons sequencing and metaproteomics analysis. Based on high-throughput amplicons sequencing analysis, the fungal genus *Saccharomycopsis* was dominant in diffused *Jiuqu* and *Pichia* was dominant in squeezed *Jiuqu* (**Supplementary Figure S2** and **Supplementary Table S3**), however, these dominant genera were poorly represented in the metaproteomics data (**Figure 3** and **Supplementary Table S6**). In contrast, the metaproteomics analysis demonstrated *Rhizopus* was the major source of identified proteins (**Figure 3A** and **Supplementary Table S6**). One possible reason could be that *Mucorales* was dominant in the early stage of starter’s fermentation and secreted most proteins, then declining in the late stage (Li et al., 2015).

Microbial members provide primary productivity to relevant ecosystem services, and local endogenous or exogenous factors may impact the structural shift of microbial communities (Perez-Izquierdo et al., 2017). In the previous study, primary endogenous factors (temperature, moisture, acidity) drive the formation of microbial structure and function (Li et al., 2016; Xiao et al., 2017). These profound understanding might help to regulate microbial communities’ formation by adjusting relevant environmental parameters. In our study, the manufacturing process change induces the variation of the major exogenous factor, leading to squeezed *Jiuqu* presented a higher volumetric weight (**Figure 1C**). This volumetric weight change induced moisture and acidity parameters variation to influence the growth of the key functional microbiota and the phenotype of enzyme activity (**Figure 5** and **Supplementary Table S8**). Our results highlighted that volumetric weight, as a primary exogenous driver, promoted the formation of the key functional microbiota in *Jiuqu*. Thus this progression might take place via endogenous factors’ driving, such as moisture, acidity parameters and so on. Further studies should focus on synergistic effect of exogenous and endogenous factors on the shaping of the key functional microbiota in *Jiuqu*.

In this study, the specific identification procedure, combining high-throughput amplicons and metaproteomics analysis, open new avenues for interesting functional insights of most concerned groups. To our knowledge, there is no report showed the detail saccharifying function of complex microbial communities in Chinese *Jiuqu*. Thus, these results strengthened the validity of association between the key functional microbiota and enzymes. Furthermore, our study provides the profound understanding to regulate microbial structure and function formation by adjusting local exogenous factors. Consequently, our findings can contribute to improve the quality of saccharifying agent and enhance the aroma characteristics of fermented foods and beverages.

## Author Contributions

BW, QW, YX, and BS designed this research, executed the experiments, and analyzed the data. BW wrote the paper.

## Conflict of Interest Statement

The authors declare that the research was conducted in the absence of any commercial or financial relationships that could be construed as a potential conflict of interest.

## Footnotes

1 http://www.uniprot.org

2 http://www.iprox.org/page/HMV006.html

3 http://www.ncbi.nlm.nih.gov/BLAST/
