# Supplementary material for: Specific Volumetric Weight-Driven Shift in Microbiota Compositions With Saccharifying Activity Change in Starter for Chinese Baijiu Fermentation
Source: Front Microbiol. 2018 Sep 28;9:2349. doi: 10.3389/fmicb.2018.02349 (PMC6172349; doi:10.3389/fmicb.2018.02349)
Supplement: Supplementary file 1 [file Data_Sheet_1.zip › Supplementary Figures and Tables.DOCX]

Supplementary Material

Specific volumetric weight-driven shift in microbiota compositions with saccharifying activity change in starter for Chinese Baijiu fermentation

Bowen Wang^1,2^, Qun Wu^1^, Yan Xu^1*^, and Baoguo Sun^2*^

^1^ Key Laboratory of Industrial Biotechnology of Ministry of Education, State Key Laboratory of Food Science and Technology, Synergetic Innovation Center of Food Safety and Nutrition, School of Biotechnology, Suqian industrial technology research institute of Jiangnan university, Jiangnan University, Wuxi, Jiangsu, China

^2^ Beijing Advanced Innovation Center for Food Nutrition and Human Health, School of Food and Chemical Engineering, Beijing Technology and Business University, Beijing, China

*** Correspondence:** Yan Xu and Baoguo Sun: yxu@jiangnan.edu.cn, sunbg@btbu.edu.cn

**This supplementary files includes:**

Supplementary Figures (Figure S1-S2)

Supplementary Tables (Table S1-S8)

**Other supplementary information for this manuscript includes the following:**

Supplementary Table S5 as an Excel file: Supplementary_Table_S5.xls in Supplementary_Material_2

# Supplementary Figures and Tables

## Supplementary Figures


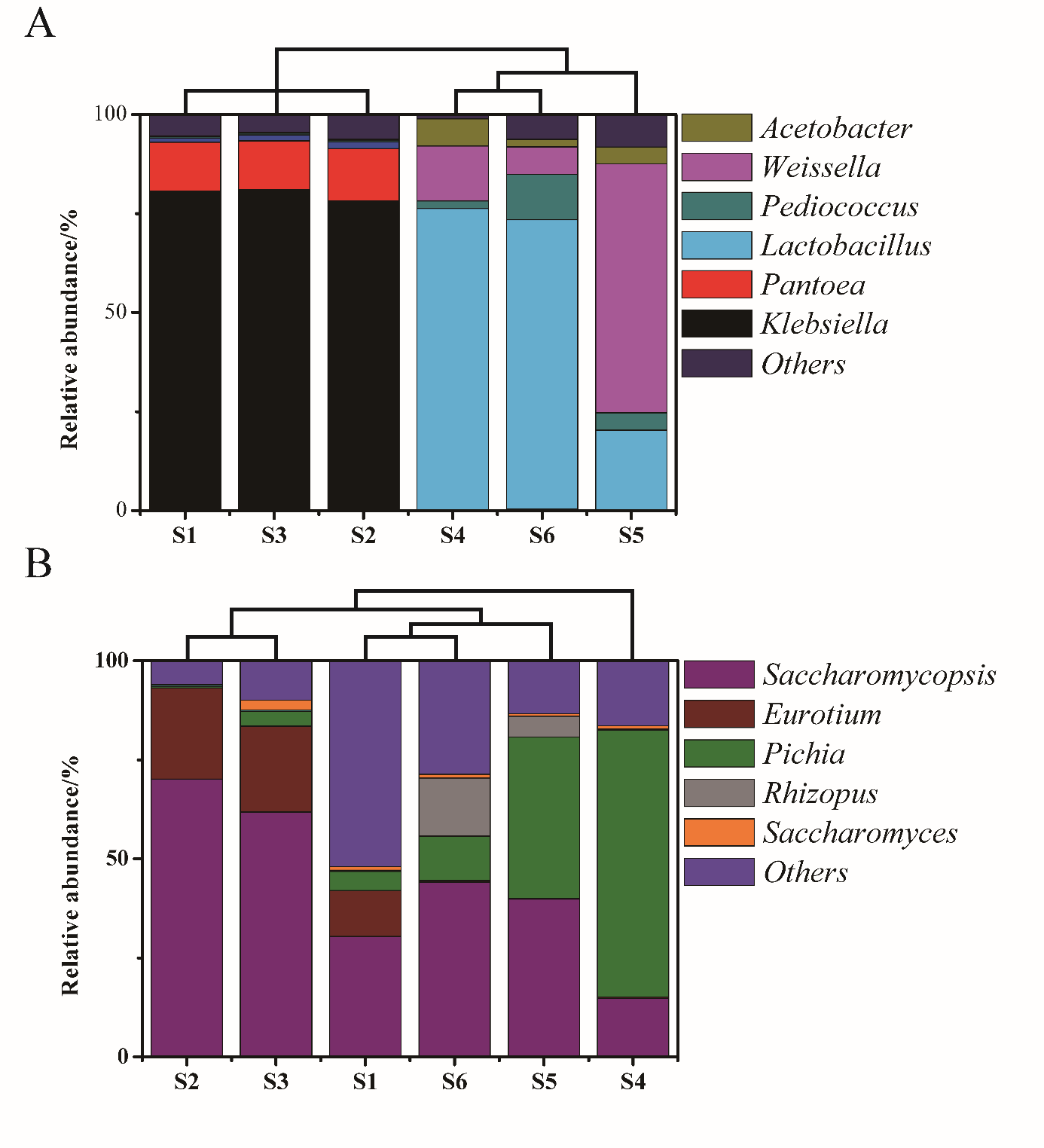


**Supplementary Figure S1.** The relative abundance of bacterial (A) and fungal (B) genera in Diffused and Squeezed *Jiuqu*. Sample 1, 2 ,3 were grouped to Diffused *Jiuqu*, and Sample 4, 5, 6 were grouped to Squeezed *Jiuqu*.


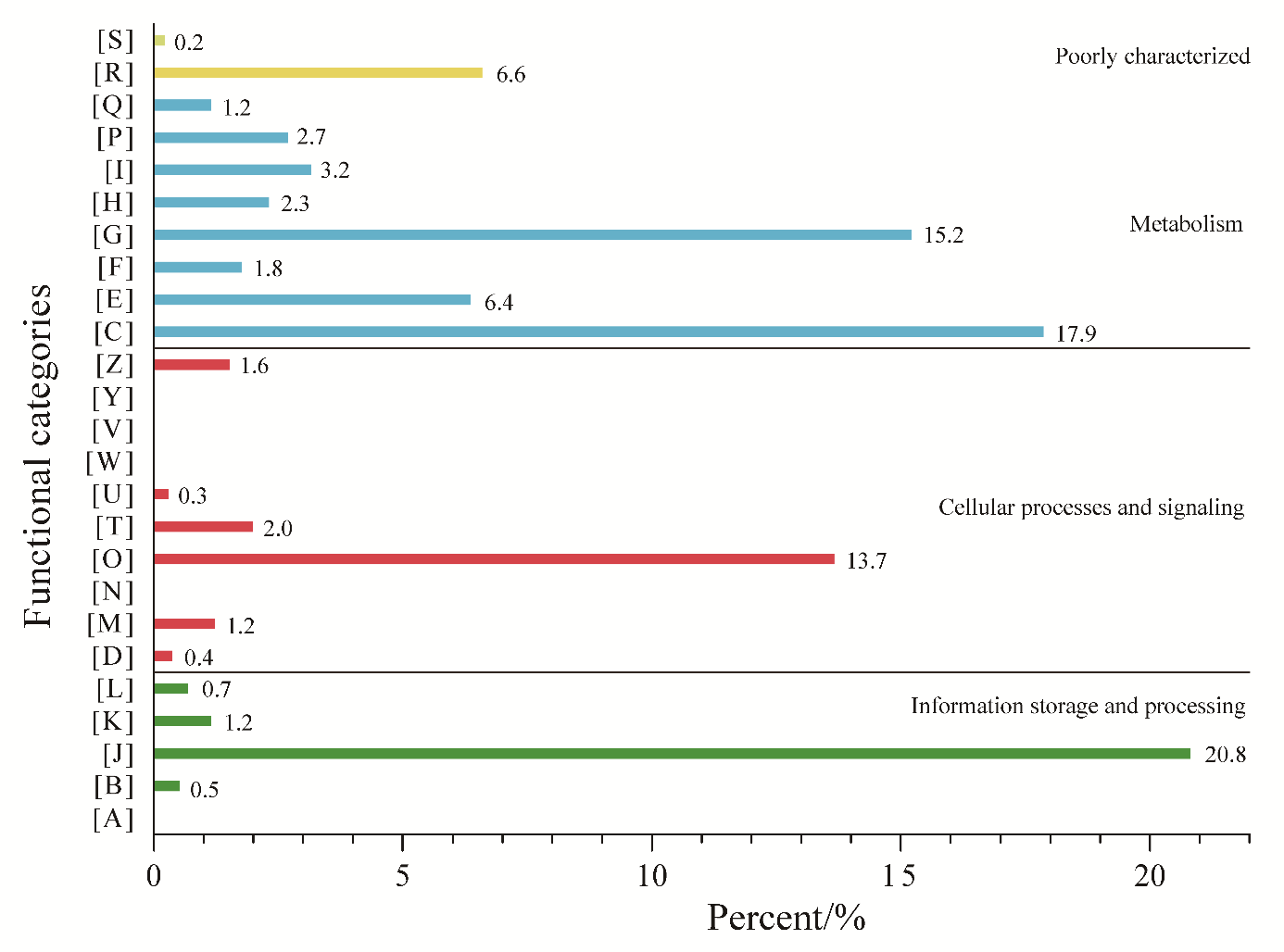


**Supplementary Figure S2.** Distribution of the identified non-redundant protein groups into Clusters of Orthologous Groups by the metaproteomics analysis. [S] Function unknown, [R] General function prediction only, [Q] Secondary metabolites biosynthesis, transport and catabolism, [P] Inorganic ion transport and metabolism, [I] Lipid transport and metabolism, [H] Coenzyme transport and metabolism, [G] Carbohydrate transport and metabolism, [F] Nucleotide transport and metabolism, [E] Amino acid transport and metabolism, [C] Energy production and conversion, [Z] Cytoskeleton, [Y] Nuclear structure, [V] Defense mechanisms, [W] Extracellular structures, [U] Intracellular trafficking, secretion and vesicular transport, [T] Signal transduction mechanisms, [O] Post-translational modification, protein turnover and chaperon functions, [N] Cell motility, [M] Cell wall/membrane/envelope biogenesis, [D] Cell cycle control, cell division, chromosome partitioning, [L] Replication, recombination and repair, [K] Transcription, [J] Translation, ribosomal structure and biogenesis, [B] Chromatin structure and dynamics, [A] RNA processing and modification.

## Supplementary Tables

**Supplementary Table S1.** Contents of Bacteria, molds, yeasts in Diffused and Squeezed *Jiuqu* by RT-qPCR analysis

| Group | Contents/lg(copies/g DW) | | |
| --- | --- | --- | --- |
|  | Bacteria | Molds | Yeasts |
| Diffused *Jiuqu* (n=3) | 5.89 ± 0.17 | 5.34 ± 0.03 | 4.27 ± 0.24 |
| Squeezed *Jiuqu* (n=3) | 6.93 ± 0.45 | 5.76 ± 0.19 | 4.76 ± 0.11 |

**Supplementary Table S2.** Bacterial and fungal microbiota diversity index based on 16S rRNA and ITS amplicons sequencing across samples

| Sample ID | Bacteria |  |  |  |  | Fungi |  |  |  |  |
| --- | --- | --- | --- | --- | --- | --- | --- | --- | --- | --- |
|  | Clean reads | OTU number | Goods' coverage | Chao1 richness | Shannon diversity index | Clean reads | OTU number | Goods' coverage | Chao1 richness | Shannon diversity index |
| S1 | 24,984 | 26 | 0.9995 | 48.0 | 1.22 | 23,463 | 137 | 0.9907 | 667.4 | 4.56 |
| S2 | 19,286 | 27 | 0.9991 | 64.6 | 1.35 | 24,303 | 21 | 0.9979 | 136.6 | 1.36 |
| S3 | 21,448 | 18 | 0.9985 | 121.0 | 1.20 | 40,785 | 69 | 0.9942 | 374.3 | 2.23 |
| S4 | 24,256 | 17 | 0.9991 | 55.1 | 1.34 | 39,897 | 91 | 0.9913 | 565.7 | 2.49 |
| S5 | 15,730 | 24 | 0.9989 | 78.0 | 1.98 | 29,150 | 72 | 0.9920 | 536.0 | 2.81 |
| S6 | 16,851 | 24 | 0.9992 | 67.2 | 1.70 | 52,291 | 156 | 0.9899 | 673.1 | 3.90 |

**Supplementary Table S3.** Relative abundances of 16S rRNA and ITS gene of *Jiuqu* with Greengene 16S rRNA and ITS gene database at the genus level

| Kingdom | Phyla/genera | Relative abundance (%) |  |
| --- | --- | --- | --- |
|  |  | Diffused *Jiuqu* | Squeezed *Jiuqu* |
| Bacteria | Firmicutes |  |  |
|  | *Lactobacillus* | 1.43 ± 0.34 | 56.49 ± 31.47 |
|  | *Weissella* | 0.14 ± 0.02 | 27.89 ± 30.48 |
|  | Proteobacteria |  |  |
|  | *Acetobacter* | 0.07 ± 0.03 | 4.31 ± 2.45 |
|  | *Acinetobacter* | 1.72 ± 0.51 | 0.09 ± 0.06 |
|  | *Klebsiella* | 79.95 ± 1.55 | 0.14 ± 0.15 |
|  | *Pantoea* | 12.61 ± 0.51 | 0.05 ± 0.07 |
|  | *Pediococcus* | 0.41 ± 0.04 | 5.89 ± 4.90 |
|  | *Rhizobium* | 0.11 ± 0.04 | 2.85 ± 2.42 |
|  | Others | 3.56 ± 0.47 | 2.28 ± 1.28 |
| Fungi | Ascomycota |  |  |
|  | *Aspergillus* | 0.63 ± 0.29 | 1.26 ± 1.88 |
|  | *Cyberlindnera* | 1.99 ± 1.31 | 0.52 ± 0.33 |
|  | *Eurotium* | 18.74 ± 6.23 | 0.35 ± 0.07 |
|  | *Fusarium* | 0.64 ± 0.79 | 1.04 ± 0.59 |
|  | *Phoma* | 0.85 ± 1.31 | 1.27 ± 0.39 |
|  | *Pichia* | 2.98 ± 2.34 | 39.79 ± 28.11 |
|  | *Saccharomyces* | 1.14 ± 1.23 | 0.79 ± 0.17 |
|  | *Saccharomycopsis* | 54.11 ± 20.95 | 32.87 ± 15.87 |
|  | Zygomycota |  |  |
|  | *Mortierella* | 0.63 ± 0.81 | 0.99 ± 0.44 |
|  | *Rhizomucor* | 0.07 ± 0.01 | 1.635 ± 1.269 |
|  | *Rhizopus* | 0.38 ± 0.11 | 6.72 ± 7.29 |
|  | Others | 17.83 ± 23.67 | 12.77 ± 4.55 |

**Supplementary Table S4.** Obtained and assigned spectrum numbers in Diffused and Squeezed *Jiuqu*

| Total Spectrum | Identified Spectrum | Peptide number | Protein number | Protein group number |
| --- | --- | --- | --- | --- |
| 343,096 | 49,799 | 10,055 | 3,973 | 1,733 |

**Supplementary Table S5.** Identified non-redundant protein groups in Diffused and Squeezed *Jiuqu* as an Excel file: Supplementary_Table_S5.xls in data sheet 2

**Supplementary Table S6.** Taxonomic distribution of the identified non-redundant protein groups in Diffused and Squeezed *Jiuqu*

| Kingdom | Phyla/genera | Relative abundance (%) | |
| --- | --- | --- | --- |
|  |  | Diffused *Jiuqu* | Squeezed *Jiuqu* |
| Bacteria | Firmicutes |  |  |
|  | *Lactobacillus* | 1.01 ± 0.08 | 4.27 ± 2.66 |
| Fungi | Ascomycota |  |  |
|  | *Aspergillus* | 3.95 ± 0.30 | 1.06 ± 0.44 |
|  | *Pichia* | 1.78 ± 0.03 | 3.82 ± 2.94 |
|  | *Saccharomyces* | 0.24 ± 0.04 | 1.76 ± 0.44 |
|  | *Zygosaccharomyces* | 1.94 ± 0.20 | 1.27 ± 0.68 |
|  | Zygomycota |  |  |
|  | *Rhizopus* | 67.80 ± 0.96 | 67.15 ± 5.56 |
|  | *Mucor* | 1.26 ± 0.07 | 1.35 ± 0.08 |
| Others |  | 22.02 ± 0.60 | 19.33 ± 6.43 |

**Supplementary Table S7.** KEGG metabolic pathways identified by non-redundant protein groups in samples (n = 6)

| KO Number | Pathway definition | Number of protein | | | |
| --- | --- | --- | --- | --- | --- |
|  |  | All | Different expressed | Up expressed  in Squeezed *Jiuqu* | Down expressed  in Squeezed *Jiuqu* |
| ko00010 | Glycolysis / Gluconeogenesis | 211 | 87 | 70 | 17 |
| ko00020 | Citrate cycle (TCA cycle) | 100 | 29 | 21 | 8 |
| ko00030 | Pentose phosphate pathway | 72 | 28 | 21 | 7 |
| ko00040 | Pentose and glucuronate interconversions | 37 | 17 | 9 | 8 |
| ko00051 | Fructose and mannose metabolism | 43 | 16 | 9 | 7 |
| ko00052 | Galactose metabolism | 22 | 8 | 6 | 2 |
| ko00053 | Ascorbate and aldarate metabolism | 26 | 15 | 9 | 6 |
| ko00061 | Fatty acid biosynthesis | 7 | 2 | 2 | 0 |
| ko00062 | Fatty acid elongation | 8 | 1 | 0 | 1 |
| ko00071 | Fatty acid degradation | 52 | 26 | 19 | 7 |
| ko00072 | Synthesis and degradation of ketone bodies | 3 |  |  |  |
| ko00130 | Ubiquinone and other terpenoid-quinone biosynthesis | 4 |  |  |  |
| ko00190 | Oxidative phosphorylation | 118 | 40 | 27 | 13 |
| ko00195 | Photosynthesis | 17 | 5 | 4 | 1 |
| ko00196 | Photosynthesis - antenna proteins | 2 |  |  |  |
| ko00220 | Arginine biosynthesis | 22 | 5 | 4 | 1 |
| ko00230 | Purine metabolism | 48 | 16 | 13 | 3 |
| ko00240 | Pyrimidine metabolism | 10 | 4 | 4 | 0 |
| ko00250 | Alanine, aspartate and glutamate metabolism | 37 | 8 | 8 | 0 |
| ko00260 | Glycine, serine and threonine metabolism | 29 | 9 | 8 | 1 |
| ko00270 | Cysteine and methionine metabolism | 44 | 15 | 12 | 3 |
| ko00280 | Valine, leucine and isoleucine degradation | 53 | 18 | 11 | 7 |
| ko00281 | Geraniol degradation | 6 | 1 | 1 | 0 |
| ko00290 | Valine, leucine and isoleucine biosynthesis | 18 | 9 | 5 | 4 |
| ko00300 | Lysine biosynthesis | 5 | 1 | 1 | 0 |
| ko00310 | Lysine degradation | 43 | 17 | 10 | 7 |
| ko00330 | Arginine and proline metabolism | 33 | 14 | 10 | 4 |
| ko00340 | Histidine metabolism | 24 | 13 | 9 | 4 |
| ko00350 | Tyrosine metabolism | 33 | 14 | 13 | 1 |
| ko00360 | Phenylalanine metabolism | 12 | 1 | 1 | 0 |
| ko00361 | Chlorocyclohexane and chlorobenzene degradation | 2 |  |  |  |
| ko00362 | Benzoate degradation | 5 |  |  |  |
| ko00364 | Fluorobenzoate degradation | 2 |  |  |  |
| ko00380 | Tryptophan metabolism | 44 | 18 | 11 | 7 |
| ko00400 | Phenylalanine, tyrosine and tryptophan biosynthesis | 10 | 2 | 1 | 1 |
| ko00410 | beta-Alanine metabolism | 36 | 15 | 9 | 6 |
| ko00430 | Taurine and hypotaurine metabolism | 5 | 2 | 2 | 0 |
| ko00450 | Selenocompound metabolism | 7 | 2 | 2 | 0 |
| ko00460 | Cyanoamino acid metabolism | 4 |  |  |  |
| ko00471 | D-Glutamine and D-glutamate metabolism | 1 |  |  |  |
| ko00480 | Glutathione metabolism | 31 | 14 | 13 | 1 |
| ko00500 | Starch and sucrose metabolism | 52 | 21 | 17 | 4 |
| ko00511 | Other glycan degradation | 1 |  |  |  |
| ko00520 | Amino sugar and nucleotide sugar metabolism | 44 | 16 | 10 | 6 |
| ko00521 | Streptomycin biosynthesis | 12 | 3 | 3 | 0 |
| ko00524 | Butirosin and neomycin biosynthesis | 3 |  |  |  |
| ko00561 | Glycerolipid metabolism | 23 | 14 | 9 | 5 |
| ko00562 | Inositol phosphate metabolism | 11 | 5 | 3 | 2 |
| ko00564 | Glycerophospholipid metabolism | 7 | 2 | 2 | 0 |
| ko00565 | Ether lipid metabolism | 3 |  |  |  |
| ko00590 | Arachidonic acid metabolism | 4 |  |  |  |
| ko00591 | Linoleic acid metabolism | 6 | 3 | 2 |  |
| ko00592 | alpha-Linolenic acid metabolism | 6 | 3 | 2 | 1 |
| ko00620 | Pyruvate metabolism | 102 | 40 | 30 | 10 |
| ko00623 | Toluene degradation | 2 |  |  |  |
| ko00625 | Chloroalkane and chloroalkene degradation | 39 | 23 | 18 | 5 |
| ko00626 | Naphthalene degradation | 17 | 12 | 11 | 1 |
| ko00627 | Aminobenzoate degradation | 8 | 3 | 2 | 1 |
| ko00630 | Glyoxylate and dicarboxylate metabolism | 74 | 22 | 15 | 7 |
| ko00640 | Propanoate metabolism | 35 | 9 | 6 | 3 |
| ko00643 | Styrene degradation | 4 |  |  |  |
| ko00650 | Butanoate metabolism | 22 | 5 | 4 | 1 |
| ko00660 | C5-Branched dibasic acid metabolism | 1 |  |  |  |
| ko00670 | One carbon pool by folate | 11 | 3 | 2 | 1 |
| ko00680 | Methane metabolism | 71 | 27 | 19 | 8 |
| ko00710 | Carbon fixation in photosynthetic organisms | 118 | 45 | 33 | 12 |
| ko00720 | Carbon fixation pathways in prokaryotes | 33 | 9 | 8 | 1 |
| ko00730 | Thiamine metabolism | 4 |  |  |  |
| ko00740 | Riboflavin metabolism | 1 |  |  |  |
| ko00750 | Vitamin B6 metabolism | 2 |  |  |  |
| ko00770 | Pantothenate and CoA biosynthesis | 12 | 7 | 4 | 3 |
| ko00780 | Biotin metabolism | 2 |  |  |  |
| ko00790 | Folate biosynthesis | 1 |  |  |  |
| ko00860 | Porphyrin and chlorophyll metabolism | 2 |  |  |  |
| ko00900 | Terpenoid backbone biosynthesis | 3 |  |  |  |
| ko00903 | Limonene and pinene degradation | 19 | 10 | 6 | 4 |
| ko00910 | Nitrogen metabolism | 13 | 4 | 4 | 0 |
| ko00920 | Sulfur metabolism | 4 |  |  |  |
| ko00970 | Aminoacyl-tRNA biosynthesis | 12 | 5 | 5 | 0 |
| ko01040 | Biosynthesis of unsaturated fatty acids | 5 | 1 | 0 | 1 |
| ko01100 | Metabolic pathways | 669 |  |  |  |
| ko01110 | Biosynthesis of secondary metabolites | 447 |  |  |  |
| ko01120 | Microbial metabolism in diverse environments | 390 |  |  |  |
| ko01130 | Biosynthesis of antibiotics | 407 |  |  |  |
| ko01200 | Carbon metabolism | 321 | 114 | 84 | 30 |
| ko01210 | 2-Oxocarboxylic acid metabolism | 60 | 18 | 12 | 6 |
| ko01212 | Fatty acid metabolism | 22 | 4 | 2 | 2 |
| ko01220 | Degradation of aromatic compounds | 17 | 12 | 11 | 1 |
| ko01230 | Biosynthesis of amino acids | 238 | 86 | 65 | 21 |
| ko02010 | ABC transporters | 2 |  |  |  |
| ko02020 | Two-component system | 16 | 4 | 2 | 2 |
| ko03008 | Ribosome biogenesis in eukaryotes | 9 | 3 | 3 | 0 |
| ko03010 | Ribosome | 206 | 78 | 51 | 27 |
| ko03013 | RNA transport | 25 | 8 | 7 | 1 |
| ko03015 | mRNA surveillance pathway | 8 | 2 | 2 | 0 |
| ko03018 | RNA degradation | 54 | 19 | 17 | 2 |
| ko03030 | DNA replication | 1 |  |  |  |
| ko03040 | Spliceosome | 43 | 13 | 8 | 5 |
| ko03050 | Proteasome | 26 | 5 | 3 | 2 |
| ko03060 | Protein export | 3 |  |  |  |
| ko03420 | Nucleotide excision repair | 1 |  |  |  |
| ko03430 | Mismatch repair | 1 |  |  |  |
| ko03440 | Homologous recombination | 1 |  |  |  |
| ko04011 | MAPK signaling pathway - yeast | 26 |  |  |  |
| ko04070 | Phosphatidylinositol signaling system | 4 |  |  |  |
| ko04111 | Cell cycle - yeast | 2 |  |  |  |
| ko04112 | Cell cycle - Caulobacter | 1 |  |  |  |
| ko04113 | Meiosis - yeast | 2 |  |  |  |
| ko04120 | Ubiquitin mediated proteolysis | 3 |  |  |  |
| ko04122 | Sulfur relay system | 4 |  |  |  |
| ko04139 | Regulation of mitophagy - yeast | 1 |  |  |  |
| ko04141 | Protein processing in endoplasmic reticulum | 74 | 35 | 23 | 12 |
| ko04144 | Endocytosis | 37 | 16 | 11 | 5 |
| ko04145 | Phagosome | 37 | 11 | 9 | 2 |
| ko04146 | Peroxisome | 36 | 16 | 11 | 5 |
| ko04213 | Longevity regulating pathway - multiple species | 51 |  |  |  |
| ko04621 | NOD-like receptor signaling pathway | 11 | 7 | 7 | 0 |

**Supplementary Table S8.** Physicochemical parameters, microbial population and glucoamylase activity in culture-dependent analysis

| ID | Volumetric weight (g/cm^3^) | Moisture (g/100 g) | Acidity (mmol/10 g DW) | Content (lg(copies/g DW)) | Glucoamylase (U/g DW) |
| --- | --- | --- | --- | --- | --- |
| 1 | 0.27 ± 0.02 | 68.73 ± 0.22 | 1.05 ± 0.07 | 6.86 ± 0.07 | 66.69 ± 15.28 |
| 2 | 0.32 ± 0.03 | 67.76 ± 0.40 | 0.87 ± 0.05 | 6.57 ± 0.06 | 165.90 ± 26.92 |
| 3 | 0.37 ± 0.06 | 67.94 ± 0.65 | 0.87 ± 0.03 | 6.65 ± 0.21 | 200.30 ± 26.92 |
| 4 | 0.46 ± 0.03 | 67.39 ± 0.48 | 0.64 ± 0.13 | 6.76 ± 0.12 | 272.70 ± 14.09 |
| 5 | 0.58 ± 0.01 | 66.93 ± 0.39 | 0.61 ± 0.07 | 7.39 ± 0.17 | 266.00 ± 21.30 |
